# Detection distribution of CNVs of *SNX29* in three goat breeds and their associations with growth traits

**DOI:** 10.3389/fvets.2023.1132833

**Published:** 2023-08-29

**Authors:** Qian Wang, Xiaoyue Song, Yi Bi, Haijing Zhu, Xianfeng Wu, Zhengang Guo, Mei Liu, Chuanying Pan

**Affiliations:** ^1^College of Animal Science and Technology, Key Laboratory of Animal Genetics, Breeding and Reproduction of Shaanxi Province, Northwest A&F University, Yangling, Shaanxi, China; ^2^Shaanxi Provincial Engineering and Technology Research Center of Cashmere Goats, Yulin University, Yulin, Shaanxi, China; ^3^Life Science Research Center, Yulin University, Yulin, Shaanxi, China; ^4^Institute of Animal Husbandry and Veterinary, Fujian Academy of Agricultural Sciences, Fuzhou, Fujian, China; ^5^Animal Husbandry and Veterinary Science Institute of Bijie City, Bijie, Guizhou, China; ^6^College of Animal Science and Technology, Hunan Agricultural University, Changsha, Hunan, China

**Keywords:** *sorting nexin 29* (*SNX29*) gene, copy number variation (CNV), growth traits, goats, marker-assisted selection (MAS)

## Abstract

As a member of the SNX family, the *goat sorting nexin 29* (*SNX29*) is initially identified as a myogenesis gene. Therefore, this study aimed to examine the polymorphism in the *SNX29* gene and its association with growth traits. In this study, we used an online platform to predict the structures of the *SNX29* protein and used quantitative real-time PCR to detect potential copy number variation (CNV) in Shaanbei white cashmere (SBWC) goats (*n* = 541), Guizhou black (GB) goats (*n* = 48), and Nubian (NB) goats (*n* = 39). The results showed that goat *SNX29* protein belonged to non-secretory protein. Then, five CNVs were detected, and their association with growth traits was analyzed. In SBWC goats, CNV1, CNV3, CNV4, and CNV5 were associated with chest width and body length (*P* < 0.05). Among them, the CNV1 individuals with gain and loss genotypes were superior to those individuals with a median genotype, but CNV4 and CNV5 of individuals with the median genotype were superior to those with the loss and gain genotypes. In addition, individuals with the gain genotype had superior growth traits in CNV3. In brief, this study suggests that the CNV of *SNX29* can be used as a molecular marker in goat breeding.

## Introduction

Members of the SNX family are located in membrane-binding cytoplasm and can bind to phosphatidylinositol via the PX domain and interact with membrane-associated protein complexes, which play an important role in regulating endocytosis and protein transport through cell membrane compartments (1, 2). To date, 32 members have been identified, and they are divided into five subgroups based on protein domain. Among them, the *SNX29* gene belongs to the SNX-PX subfamily (3), which has been reported to be involved in disease, nervous system development, and animal growth. Studies have linked the *SNX29* gene to schizophrenia (SCZ), autism, and other psychiatric disorders (4, 5). The deletion of *SNX29* intron 14 may lead to primary testicular lymphoma (6). Zhu et al. found that downregulation of the *SNX29* gene was associated with epithelial ovarian carcinoma cells (7). Furthermore, Sparks et al. showed a strong association between IgA levels and the region between 6.89 and 14.95 Mb on sheep chromosome 24, which corresponds to the *SNX29* gene (8). A circRNA of the *SNX29* gene regulated the proliferation and differentiation of muscle cells (9). Studies have shown that the *SNX29* gene plays a key role in subcutaneous fat deposition in Xiangdong black (XDB) goats, and the *SNX29* CNV is significantly associated with the chest and abdominal girth of XDB goats (*P* < 0.01) (10). Based on the above, the *SNX29* gene was selected to be studied in this study.

Copy number variation (CNV) exists widely in the genomes of organisms, and it is considered to be an important source of genetic differences between individuals (11, 12). In recent years, some studies reported that CNV was significantly correlated with the economic traits of livestock, such as litter size (13), meat quality (14), milk production (15), weight gain rate (16), and feed conversion rate (17). The advantages of CNV-promoting population diversity, simplicity, and efficiency were discovered by more people (18). As a applicable molecular marker, CNV can make marker-assisted selection (MAS) better play the advantages of convenience, simplicity, and so on. In short, it provides new ideas and methods for breeding work.

Shaanbei white cashmere (SBWC) goats were bred from Liaoning white cashmere goat and Ziwuling black goat (19), which has high cashmere value and meat value (20). Guizhou black (GB) goats are an excellent local breed with good meat quality and coarse feeding tolerance (21). Nubian (NB) goats have good value for meat and milk and have higher meat content than other dual-purpose goats (22). However, their growth performance fails to achieve the expected results, so it is helpful to increase the economic value of goats by improving their growth traits through MAS.

Currently, the CNVs of the *SNX29* gene and its association with growth traits in SBWC goats have not been reported. Therefore, this study is characterized based on the aspects of protein structure, physicochemical properties, and DNA variation. Next, we explored five potential CNVs, which were detected in SBWC goats, GB goats, and NB goats by quantitative real-time PCR (qRT-PCR). An association analysis was carried out between the *SNX29* gene and the growth traits of goats. These results will have a deeper understanding of gene variation and livestock growth traits, in order to lay a theoretical foundation for MAS breeding of goats.

## Materials and methods

### Animal welfare explanation

The samples used in this experiment comply with the Regulations on the Administration of Experimental Animals at Northwest A&F University (NWAFU-314020038).

### Prediction of *SNX29* protein physicochemical properties and structure

Using NCBI-searched *SNX29* protein sequences, the goats' *SNX29* protein amino acid number, molecular weight, and isoelectric point were calculated using the Expasy online platform, and the ProtScale application and ProtParam were used to predict the protein hydrophobicity. The *SNX29* protein of transmembrane signal peptide was predicted using the TMHMM database and SignalP 4.1. The AlphaFold and SOPMA online platforms were used to predict the advanced structure of the *SNX29* protein (23) (Supplementary Table S1).

### Sample collection and genomic DNA extraction

Under the same feeding conditions, ear tissues of 541 SBWC goats, 48 GB goats, and 39 NB goats were selected from the Yulin goat farm in Shaanxi province, the Bijie goat farm in Guizhou province, and the Zhangzhou Nubian goat breeding cooperative in Fujian province. All the individuals were female goats (2–3 years) and were not related to each other. Genomic DNA was extracted from goat ear tissue using the high salt extraction method (24) and stored at 70% alcohol at −80°C (25). A NanoDrop™2000 spectrophotometer (Thermo Scientific, Waltham, MA, USA) was used to measure the OD_260/280_ ratio, and a ratio between 1.8 and 2.0 means that the nucleic acid concentration is qualified (26). Then, the extracted DNA was placed at −40°C.

### Primer designing

We searched the Animal Omics database (27) (Supplementary Table S1) and found five CNV loci of the *SNX29* gene in goats. Five pairs of amplified primers were referenced in a previous article (28).

### CNV genotyping detection of the *SNX29* gene

To ensure that the primers can amplify the target fragment, the primers are detected through the mixed pool (CNV1 = 137 bp, CNV2 = 138 bp, CNV3 = 104 bp, CNV4 = 151 bp, and CNV5 = 109 bp). Next, 541 SBWC goat samples, 48 GB goat samples, and 39 NB goat samples were used to detect the CNV loci. qRT-PCR amplification systems and procedures refer to previous laboratory articles (29, 30). The result was processed using method 2 ^*^2(−δCt) (31).

### Statistical analyses

The association between the variants and growth traits was explored using the analysis of variance (ANOVA) and independent sample *t*-test in SPSS 26.0 (IBM, USA), and the chi-square (χ^2^) test was used to analyze the significance between the three breeds (32). And the line model was used as a reference by Liu et al. (33). Where Y_ijk_ = α_i_ + β_j_ + e_ijk_ + u acts as an analysis model, Y_ijk_ is the evaluation of growth traits at the i level of fixed factor age (α_i_) and j level of fixed factor genotype (β_j_), u is the overall mean, and e_ijk_ is the random error.

## Results

### Prediction of *SNX29* protein physicochemical properties and structure

**Figure 1:**
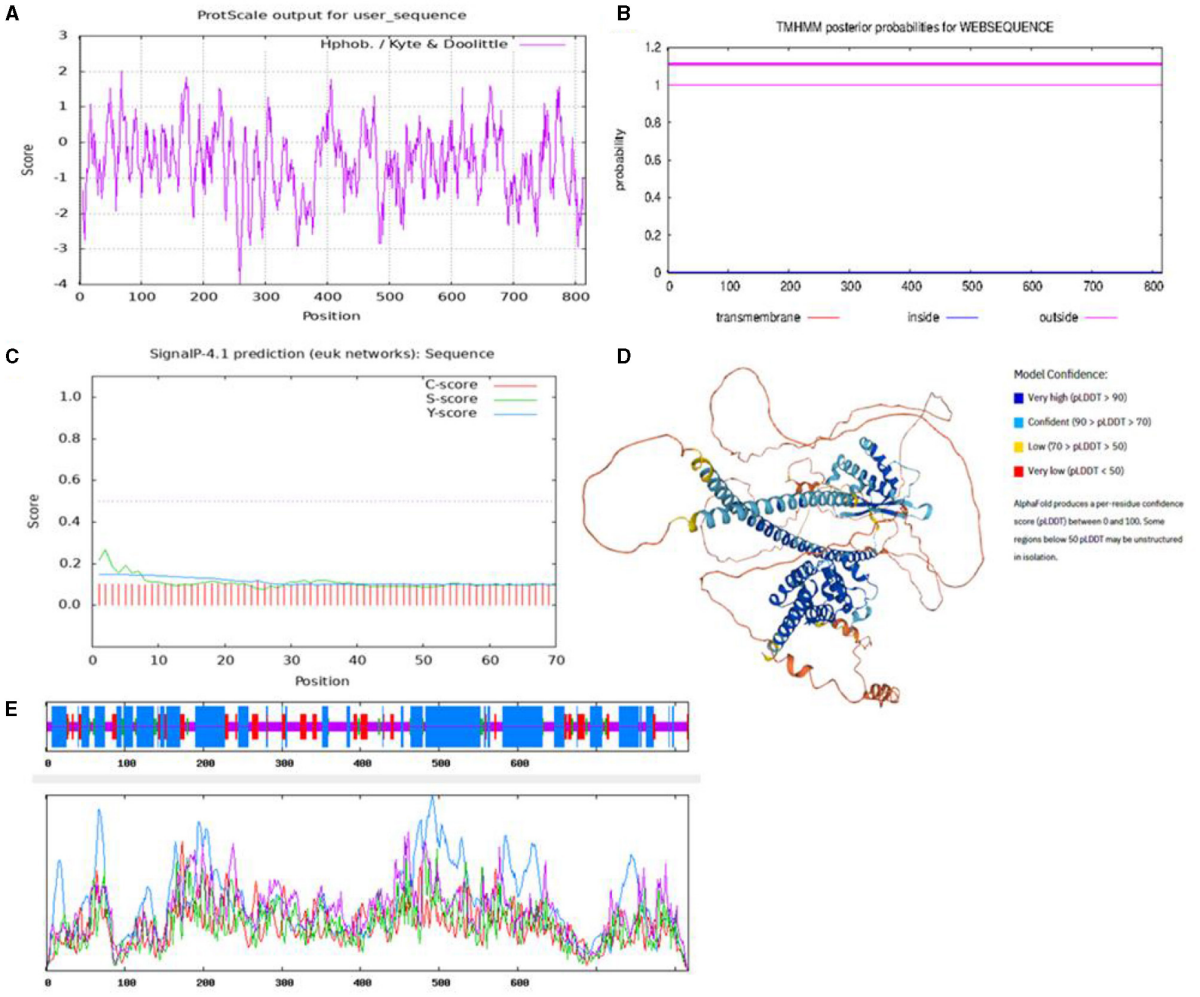
Prediction of physicochemical properties and structure of the *SNX29* protein. **(A)** Hydrophobicity of goat *SNX29* protein. **(B)** Goat *SNX29* protein transmembrane signal peptide. **(C)** Goat *SNX29* protein transmembrane signal peptide prediction. The abscissa axis represents the sequence number of amino acid residues corresponding to the submitted protein sequence; the value of the ordinate axis is the probability value of each amino acid located on the inside, outside, and TMhelix on the abscissa axis. **(D)** Three-dimensional model of goat *SNX29* protein tertiary structure. **(E)** Secondary structural parameters of goat *SNX29* protein. Blue means a-helix, red means extended backbone, green means β-folding, and yellow means random crimping.

To characterize the functions of the *SNX29* gene, the protein structure and physicochemical properties were predicted. The results showed that the protein contained 817 amino acids, the molecular weight was 9,143.14, and the isoelectric point was 5.90 by the Expasy online platform. ProtScale online software predicted the hydrophobicity of the protein, and the results showed that there were more hydrophilic residues in the goat *SNX29* protein, which indicated that this protein was hydrophilic (Figure 1A). The results were consistent with ProtParam online software predictions. TMHMM prediction results showed that the protein encoded by the *SNX29* gene did not have transmembrane helix (Figure 1B). SignalP 4.1 prediction results showed that the D critical value of signal peptide and non-signal peptide of this protein was 0.450, and the D critical value of the *SNX29* protein was 0.155 (Figure 1C). According to the signal peptide hypothesis, the *SNX29* protein had no signal peptide and belonged to non-secretory protein. The SOPMA online platform predicted the detailed information on the secondary structure of *SNX29* protein, and the results showed that alpha helix accounted for 47.98%, extended strand accounted for 12.24%, β-turn accounted for 4.04%, and random coil accounted for 35.74% (Figure 1E). AlphaFold online software predicted the three-dimensional structure of the *SNX29* protein (Figure 1D).

### Frequency of CNV genotypes in goats

**Figure 2:**
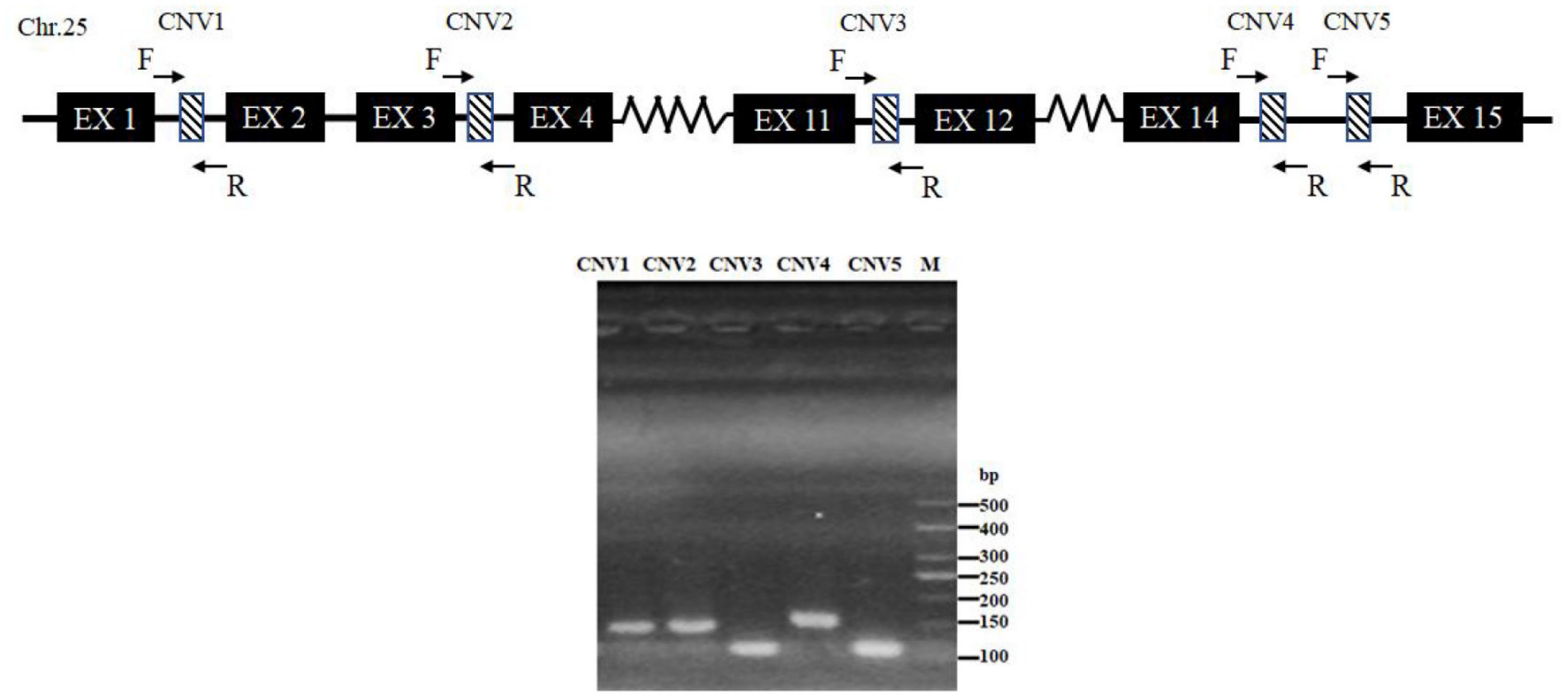
Schematic diagram of the PCR assay for CNVs of the goat *SNX29* gene. Chr, chromosome; EX, exon; F, forward primer; R, reverse primer; M, means DNA marker.

**Figure 3:**
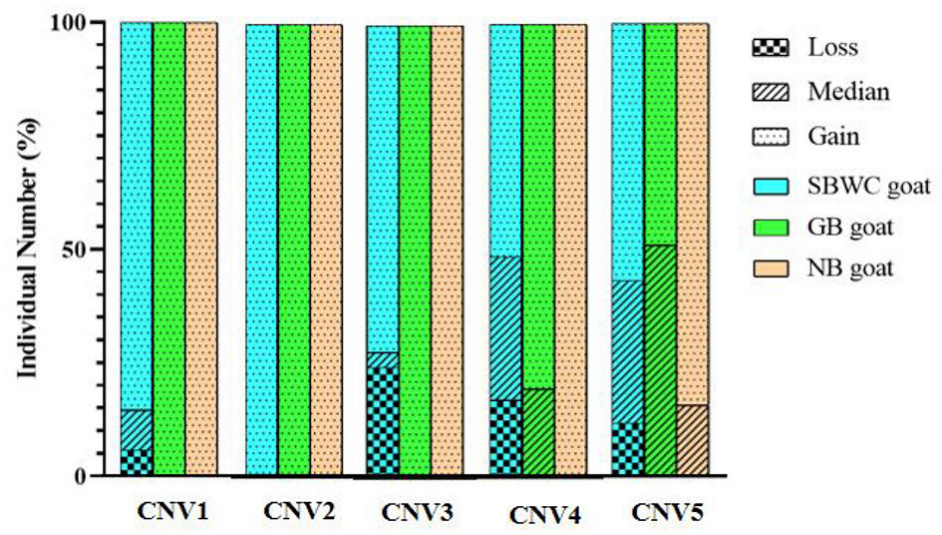
Genotyping proportion of CNVs in SBWC goat, GB goat, and NB goat. In CNV1: the total individual number of SBWC goats was 278, GB goats was 48, and NB goats was 38; in CNV2: the total individual number of SBWC goats was 290, GB goats was 48, and NB goats was 39; in CNV3: the total individual number of SBWC goats was 363, GB goats was 48, and NB goats was were 281, GB goats was 46, and NB goats was 38; in CNV5: the total individual number of SBWC goats was 372, GB goats was 48, and NB goats was 39.

After mixed pool detection, it was found that the five CNVs were consistent with the target band (Figure 2). Then, by expanding the sample size for testing, the following results were obtained: In CNV1, the proportion of gain genotype was greater than that of median and loss genotypes in goats. There were 85.61% individuals with gain genotypes in the SBWC goats; however, and all individuals in the GB goats and NB goats were gain genotypes; in CNV2, all three goat breeds were gain genotype; in CNV3, there were 72.18% individuals of gain genotype, 3.31% individuals of median genotype, and 24.52% individuals of loss genotype in SBWC goats, and all GB goats and NB goats were gain genotype; in CNV4, there were 51.25% individuals of gain genotype, 31.67% individuals of median genotype, and 17.08% individuals of loss genotype in SBWC goats, there were 80.43% individuals of gain genotype, 19.57% individuals of median genotype in GB goats, and NB goats were all gain genotype; and in CNV5, there were 56.72% individuals of gain genotype, 31.45% individuals of median genotype, and 11.83% individuals of loss genotype in SBWC goats, there were 48.94% individuals of gain genotype, 51.06% individuals of median genotype in GB goats, there were 84.21% individuals of gain genotype, and 15.79% individuals of median genotype in NB goats (Figure 3).

### Association analysis between CNVs and the goat *SNX29* gene

The association analysis results showed that four CNVs were related to growth traits in SBWC goats. CNV1 was significantly associated with chest width (*P* = 0.002), body length (*P* = 1.230E-4), body height (*P* = 0.008), cannon circumference (*P* = 1.300E-5), and heart girth (*P* = 0.033). CNV3 was significantly associated with chest width (*P* = 0.004) and cannon circumference (*P* = 0.009). CNV4 was significantly associated with chest width (*P* = 8.166E-7), heart girth (*P* = 2.620E-4), and cannon circumference (*P* = 0.001). CNV5 was significantly associated with chest depth (*P* = 0.008) and body length (*P* = 0.025). Additionally, in the association analysis between growth traits of SBWC goats and CNVs, we found that in CNV1 individuals, gain and loss genotypes were superior to those with median genotype on the aspect of growth traits, but in CNV4 and CNV5 individuals, median genotypes were superior to loss and gain. In addition, in the CNV3, the gain genotype performed better growth traits (Table 1). The χ^2^ test results showed that except for CNV2, the remaining CNV loci were significantly associated among the SBWC goats, GB goats, and NB goats (*P* < 0.01) (Table 2).

**Table 1:** Association analysis between growth traits and the CNVs in SBWC goats.

**CNV loci**	**Trait types**	**Typical frequencies (AVG** ±**SE)**			***P*-values**
		**Loss**	**Median**	**Gain**	
CNV1	Height at hip cross (cm)	60.84 ± 0.99 (*n* = 16)	58.60 ± 0.80 (*n* = 24)	60.75 ± 0.29 (*n* = 235)	0.066
	**Chest width (cm)**	**21.28** **±0.95**^**A**^ **(*****n*** **=** **16)**	**18.04** **±0.40**^**B**^ **(*****n*** **=** **24)**	**20.05** **±0.20**^**A**^ **(*****n*** **=** **235)**	**0.002**
	Chest depth (cm)	29.56 ± 0.63^Ab^ (*n* = 16)	28.40 ± 0.34^b^ (*n* = 24)	29.69 ± 0.20^A^ (*n* = 235)	0.135
	**Body length (cm)**	**65.34** **±0.88**^**AB**^ **(*****n*** **=** **16)**	**63.65** **±0.53**^**B**^ **(*****n*** **=** **24)**	**66.49** **±0.34**^**A**^ **(*****n*** **=** **233)**	**1.230E-4**
	**Cannon circumference (cm)**	**7.98** **±0.13**^**B**^ **(*****n*** **=16)**	**7.75** **±0.10**^**B**^ **(*****n*** **=** **24)**	**8.39** **±0.05**^**A**^ **(*****n*** **=** **236)**	**1.300E-5**
	**Heart girth (cm)**	**88.38** **±1.60**^**a**^ **(*****n*** **=** **16)**	**83.15** **±1.12**^**b**^ **(*****n*** **=** **24)**	**86.69** **±0.46**^**a**^ **(*****n*** **=** **236)**	**0.033**
	**Body height (kg)**	**60.16** **±0.96**^**A**^ **(*****n*** **=** **16)**	**56.08** **±0.79**^**B**^ **(*****n*** **=** **24)**	**58.22** **±0.27**^**A**^ **(*****n*** **=** **235)**	**0.008**
CNV3	Height at hip cross (cm)	59.98 ± 0.74 (*n* = 23)	60.08 ± 1.24 (*n* = 12)	60.75 ± 0.26 (*n* = 262)	0.625
	**Chest width (cm)**	**18.78** **±0.44**^**AB**^ **(*****n*** **=** **23)**	**17.46** **±0.61**^**B**^ **(*****n*** **=** **12)**	**20.03** **±0.19**^**A**^ **(*****n*** **=** **263)**	**0.004**
	Chest depth (cm)	30.48 ± 0.88 (*n* = 23)	29.71 ± 1.00 (*n* = 12)	29.22 ± 0.16 (*n* = 263)	0.109
	Body length (cm)	66.44 ± 0.90 (*n* = 23)	65.29 ± 1.42 (*n* = 12)	66.54 ± 0.28 (*n* = 261)	0.649
	**Cannon circumference (cm)**	**7.87** **±0.12**^**B**^ **(*****n*** **=22)**	**7.92** **±0.16**^**AB**^ **(*****n*** **=** **12)**	**8.31** **±0.05**^**A**^ **(*****n*** **=** **262)**	**0.009**
	Heart girth (cm)	89.65 ± 1.51 (*n* = 22)	87.71 ± 2.09 (*n* = 12)	88.81 ± 0.51 (*n* = 262)	0.795
	Body height (kg)	60.84 ± 0.48 (*n* = 89)	56.96 ± 0.93 (*n* = 12)	58.21 ± 0.24 (*n* = 262)	0.552
CNV4	Height at hip cross (cm)	60.63 ± 0.55 (*n* = 48)	60.84 ± 0.48 (*n* = 89)	60.39 ± 0.37 (*n* = 143)	0.742
	**Chest width (cm)**	**18.08** **±0.30**^**c**^ **(*****n*** **=** **48)**	**20.50** **±0.34**^**A**^ **(*****n*** **=** **89)**	**19.39** **±0.25**^**b**^ **(*****n*** **=** **144)**	**8.166E-7**
	Chest depth (cm)	29.68 ± 0.51 (*n* = 48)	29.44 ± 0.31 (*n* = 89)	29.14 ± 0.23 (*n* = 144)	0.503
	Body length (cm)	65.27 ± 0.69 (*n* = 48)	66.64 ± 0.51 (*n* = 89)	66.51 ± 0.39 (*n* = 144)	0.223
	**Cannon circumference (cm)**	**7.90** **±0.10**^**B**^ **(*****n*** **=47)**	**8.35** **±0.08**^**A**^ **(*****n*** **=** **89)**	**8.23** **±0.06**^**A**^ **(*****n*** **=** **143)**	**0.001**
	**Heart girth (cm)**	**88.42** **±1.25**^**b**^ **(*****n*** **=** **47)**	**91.61** **±0.87**^**A**^ **(*****n*** **=** **89)**	**87.12** **±0.66**^**b**^ **(*****n*** **=** **143)**	**2.620E-4**
	Body height (kg)	58.05 ± 0.55 (*n* = 48)	58.05 ± 0.42 (*n* = 89)	58.00 ± 0.35 (*n* = 144)	0.996
CNV5	Height at hip cross (cm)	60.33 ± 0.60 (*n* = 44)	60.21 ± 0.42 (*n* = 116)	60.50 ± 0.29 (*n* = 212)	0.837
	Chest width (cm)	19.40 ± 0.50 (*n* = 44)	20.16 ± 0.26 (*n* = 116)	19.82 ± 0.23 (*n* = 212)	0.360
	**Chest depth (cm)**	**28.75** **±0.44**^**B**^ **(*****n*** **=** **44)**	**29.84** **±0.20**^**A**^ **(*****n*** **=** **116)**	**28.95** **±0.19**^**B**^ **(*****n*** **=** **212)**	**0.008**
	**Body length (cm)**	**64.59** **±0.78**^**B**^ **(*****n*** **=** **43)**	**66.85** **±0.38**^**a**^ **(*****n*** **=** **116)**	**65.93** **±0.35**^**aB**^ **(*****n*** **=** **211)**	**0.025**
	Cannon circumference (cm)	8.08 ± 0.09 (*n* = 43)	8.32 ± 0.06 (*n* = 117)	8.18 ± 0.05 (*n* = 211)	0.097
	Heart girth (cm)	90.01 ± 1.18 (*n* = 43)	87.74 ± 0.69 (*n* = 117)	87.83 ± 0.56 (*n* = 211)	0.220
	Body height (kg)	57.92 ± 0.66 (*n* = 44)	57.86 ± 0.35 (*n* = 117)	57.83 ± 0.29 (*n* = 211)	0.990

Values with different letters (A, B, C/a, b, c) within the same row differ significantly at *P* < 0.01/*P* < 0.05. AVG, means average; SE, means standard error. The bold values indicate the value of *P* < 0.05.

**Table 2:** Genotype distribution among the SBWC goats, GB goats, and NB goats.

**CNV Loci**	**Breeds**	**Size**	**Genotypic frequencies**			**χ^2^**	***P*-value**
			**Loss**	**Median**	**Gain**		
CNV1	SBWC	276	16	24	236	14.012	**0.007**
	GB	48	0	0	48		
	NB	38	0	0	38		
CNV2	SBWC	291	0	2	289	0.587	0.746
	GB	47	0	0	47		
	NB	38	0	0	38		
CNV3	SBWC	363	89	12	262	30.873	**3.000E-6**
	GB	48	0	0	48		
	NB	38	0	0	38		
CNV4	SBWC	281	48	89	144	44.819	**4.335E-9**
	GB	46	0	9	37		
	NB	38	0	0	38		
CNV5	SBWC	372	44	117	211	23.749	**9.000E-5**
	GB	47	0	24	23		
	NB	38	0	6	32		

SBWC, Shaanbei white cashmere goats; GB, means Guizhou black goats; NB, Nubian goats. The bold values indicate the value of *P* < 0.05.

## Discussion

Relevant studies have shown that *SNX7* (34, 35), *SNX8* (36), *SNX9* (37), *SNX10* (38), and *SNX19* genes (39) were associated with animal growth traits. As a member of the same family, we speculated that the *SNX29* CNVs may have a remarkable influence on growth traits. To preliminarily explore the function of the *SNX29* gene, the goat *SNX29* protein structure was predicted using an online platform. The results showed that the *SNX29* protein was hydrophilic and had no transmembrane helix and signal peptide, and it is a non-secretory protein and performed a relevant function in the cytoplasm, which was consistent with the previous description (40).

To further explore the relationship between this gene and growth traits, we conducted population validation. Five CNVs were retrieved from the database. After population distribution detection, it was found that the genotypes of goats of the three breeds were different at different loci. This is because genetic variations vary from breed to breed (41). In the three goat breeds, more individuals performed gain genotype. This may be because the gain genotype showed better economic efficiency and was retained in artificial selection. Notably, the association analysis showed that four CNVs were observably associated with chest width, body length, body height, cannon circumference, and chest circumference (*P* < 0.05) in SBWC goats, which supports our conjecture. Moreover, we found that in CNV1 individuals, the gain and loss genotypes were superior to those with the median genotypes in terms of growth traits, but in CNV4 and CNV5 individuals, the median genotypes were better than the loss and gain genotypes. In addition, in the CNV3, the gain genotype performed better growth traits, which could be due to mutation, selection, gene recombination, and genetic drift migration (42). These outcomes suggest that the gain/loss genotype of CNV1, the gain genotype of CNV3, and the median genotype of CNV4 and CNV5 have a positive effect on growth traits (43).

In this study, we found that the CNVs of *SNX29* were associated with the growth traits of goats, which is consistent with the function of *SNX29* in previous studies associated with growth. A genome-wide scan identified the growth-related SNP markers of *SNX29* in Chinese Wagyu cattle (44). Genome-wide association analysis showed that CNV27 of the *SNX29* gene was associated with growth traits of African goats (45), and also two InDels within this gene are significantly correlated with chest width, hip width, and other growth traits in goats (46). In addition, this gene has shown growth-related functions in different species. In York pigs, genome-wide association analysis of five meat quality traits found that 12 intron SNPs of the *SNX29* gene were associated with intramuscular fat content (47). Therefore, the *SNX29* has been identified as a candidate gene associated with growth traits, whose CNVs can also act as an influence on the growth traits of livestock. We will continue to explore the molecular mechanism between this gene and growth traits in further studies.

## Conclusion

In this study, the growth effect of the *SNX29* gene was elucidated from the aspects of protein structure, physicochemical properties, and DNA variation. The protein encoded by *SNX29* was a non-secreted protein, whose five CNVs were identified in SBWC goats, GB goats, and NB goats. Moreover, CNVs were found to be associated with growth traits in SBWC goats. The CNV1, CNV3, CNV4, and CNV5 were significantly associated with the SBWC goats, GB goats, and NB goats (*P* < 0.01). Thus, the *SNX29* gene may be an essential functional candidate gene for growth traits.

## Data availability statement

The original contributions presented in the study are included in the article/Supplementary material, further inquiries can be directed to the corresponding authors.

## Ethics statement

The animal study was reviewed and approved by Northwest A&F University. Written informed consent was obtained from the owners for the participation of their animals in this study.

## Author contributions

XS, HZ, XW, ZG, and CP: sample collection. QW and YB: experimental operation. QW, YB, HZ, XW, and ZG: data collation and analysis. QW: article writing. QW, YB, and CP: manuscript revision and editing. ML and CP: project management. All authors contributed to the article and approved the submitted version.
