# Supplementary material for: Detection distribution of CNVs of SNX29 in three goat breeds and their associations with growth traits
Source: Front Vet Sci. 2023 Aug 29;10:1132833. doi: 10.3389/fvets.2023.1132833 (PMC10495836; doi:10.3389/fvets.2023.1132833)
Supplement: Supplementary file 1 [file Table_1.doc]

**Table S1.** Online software used for analysis.

| Software names | URL | Purposes |
| --- | --- | --- |
| NCBI | https://www.ncbi.nlm.nih.gov/ | Sequences blast |
| Expasy | https://web.expasy.org/ | Protein physicochemical properties prediction |
| Protscale | https://web.expasy.org/protscale/ | Protein hydrophobicity prediction |
| Protparam | https://web.expasy.org/protparam/ | Protein hydrophobicity prediction |
| TMHMM | http://www.cbs.dtu.dk/services/TMHMM/ | Transmembrane signal peptide prediction |
| SignalP 4.1 | http://www.cbs.dtu.dk/services/SignalP/ | Transmembrane signal peptide prediction |
| Alphafold | https://alphafold.ebi.ac.uk/ | Protein structure prediction |
| SOPMA | http://npsa-pbil.ibcp.fr/cgi-bin/npsa_automat.pl?page=npsa_sopma.html | Protein structure prediction |
| Animal Omics | http://animal.nwsuaf.edu.cn/ | Variants found |
